# From the Basic Psychological Needs Satisfaction to Intrinsic Motivation: Mediating Effect of Academic Integration

**DOI:** 10.3389/fpsyg.2021.612023

**Published:** 2021-05-28

**Authors:** Jorge Vergara-Morales, Milenko Del Valle

**Affiliations:** ^1^Facultad de Salud y Ciencias Sociales, Universidad de Las Américas, Concepción, Chile; ^2^Departamento de Ciencias Sociales, Universidad de Antofagasta, Antofagasta, Chile

**Keywords:** basic psychological needs, intrinsic motivation, academic integration, self-determination theory, mediation analysis

## Abstract

The studies show a positive and direct relationship between basic psychological needs satisfaction and intrinsic motivation of the students. However, there is a lack of studies that analyze the psychological processes that affect these relationships. For this reason, the purpose of this study was to investigate the mediating role of academic integration on the relationship between basic psychological needs satisfaction and intrinsic motivation of Chilean university students. The participants were a total of 580 students from a university in northern Chile, 359 women and 221 men. The mediation analysis was performed by structural equations modeling, using the maximum likelihood method and the bootstrapping procedure with 10,000 iterations. The results indicated that academic integration partially mediated the relationship between basic psychological needs satisfaction and intrinsic motivation of the students. Therefore, the basic psychological needs satisfaction had an indirect effect on the intrinsic motivation of the students through academic integration. It is concluded that the academic integration constitutes a psychological process that promotes the development of intrinsic motivation for learning. The practical implications are discussed along with the limitations of the study and recommendations for future research.

## Introduction

Currently, there is empiric evidence that demonstrate not only the relevance of transition experienced by students when moving from high school to higher education but also the relevance of different factors influencing this important process that affects the development of learning (Soares et al., 2009, 2011; Fernández-Hileman et al., 2014; Borzone, 2017; Armijo et al., 2019). It is recognized that this transition stage constitutes an important milestone in the life of every student, and it is assumed as a complex process where multiple factors interact. Students are exposed to face personal, family, social, environmental, and academic challenges (Becerra-González and Reidl, 2015; Álvarez and López, 2017). In Chile, as in other countries, this process is a subject of general interest among specialists because it demonstrated the direct and significant influence of diverse factors in the learning process and their relation to success or failure in higher education (Catalán and Santelices, 2014; Garcés and Arriagada, 2015; van Rooij et al., 2018) or because those factors generate a series of personal, social, and economic consequences that affect the structure of the higher education system itself and the development of advanced human capital (Ayala and Atencio, 2018).

There is enough evidence to support that motivational variables or specific disciplinary areas are fundamental to understand the general academic results and to explain the learning process (Miñano and Castejón, 2011; Rosário, 2012; Alonso-Tapia et al., 2014; Cerda et al., 2017). Thus, it is necessary and indispensable to move forward toward the study of psychological processes that can modify the relationships established among the different motivational factors affecting the development of learning.

### Basic Psychological Needs and Intrinsic Motivation

The self-determination theory (SDT) proposed by Deci and Ryan (2000) is mainly focused on the study of biological, social, and cultural conditions supporting or thwarting the growth and psychological well-being of individuals, either in general or specific contexts (Ryan and Deci, 2017). This proposal assumes that human motivation is guided by the development of activities that people freely choose, without expecting getting rewarded. Thus, they operate from their own will assuming a personal behavior in relation to the different activities they do without the need of external pressure (Reeve and Lee, 2019). The SDT acknowledges that there are three basic psychological needs: the need for autonomy, the need for competition, and the need for relatedness. All of them are crucial for motivation and are considered essential nutrients for psychological growth, integration, and general well-being. Therefore, their satisfaction allows an efficient functioning and good health (Milyavskaya and Koestner, 2011; Del Valle et al., 2018).

The need for autonomy refers to the experimentation of behaviors as volunteers and self-governing by the individual himself. It implies internal acceptation and engagement toward the conduct motivated. Thus, it is recognized that there is a regulation of the “self.” The need for competence is related to the efficacy and the skills of the individual, in other words, when a person experiences, expresses, and develops his/her capacities and feels competent. Finally, the need for relatedness arises when a person feels important and connected to others and has a sense of belonging and relates to others from authenticity. According to several research studies, it has been demonstrated that these basic psychological needs are present in different fields such as education, work, sports, upbringing, etc., and their satisfaction is considered essential to the development of a balanced “self” (Deci and Ryan, 2008; Tian et al., 2014; Vansteenkiste et al., 2020).

Moreover, the SDT pays attention and stresses the relevance of the different types of motivation affecting and influencing people’s behavior. This theory states that motivation is not a unitary phenomenon because there are ways of motivation that belong to will and others that respond to factors, which are external to the will of individuals, as well as it recognizes different effects produced on people (Ryan and Deci, 2017). The different types of motivations can be located on a continuum gradient that implies different types of behavior regulation according to the perceived locus of causality: (a) intrinsic, which is characterized by behaviors regulated on the basis of the interest and inherent pleasure related to the development of the academic activities; (b) identified, which occurs when students value and consider the development of academic activities personally important; (c) introjected, characterized by behaviors based on the avoidance of feelings of guilt or shame or on the exaltation of the ego; and (d) extrinsic, characterized for behaviors aimed at obtaining rewards or avoiding punishment (Maulana et al., 2016; Howard et al., 2017; Litalien et al., 2017). Each motivation type is configurated in three generalization levels: a global level, which refers to an orientating, general and steady motivation, related to the personality of individuals; a contextual level, which refers to specific contexts in which a person operates—education, work, sports upbringing, etc.—and where factors such as the social ones have a strong influence; finally, a situational level, which refers to specific moments in which a person is, and they are unrepeated in time (Lavigne and Vallerand, 2010).

Regarding intrinsic motivation, it “refers to the spontaneous tendency to seek out novelty and challenges, to extend and exercise one’s capacity, to explore, and to learn […] When intrinsically motivated, people engage in an activity because they find it interesting and inherently satisfying” (Di Domenico and Ryan, 2017, p. 1). In the field of education, intrinsic motivation is observed when the students choose “to engage in an activity for its own sake, whether for interest, pleasure or satisfaction” (Wang et al., 2019, p. 2). When students are intrinsically motivated, they tend to show high levels of implication in their learning process, and they show greater engagement and persistence to achieve the learning goal (Ryan and Deci, 2020). Hence, the importance of promoting educational policies centered on the intrinsic interest of students from the perspective of the joy of learning is recognized (Wang et al., 2017). Studies show that the basic psychological needs satisfaction is positively related to intrinsic motivation (Pulyaeva and Nevryuev, 2020; Walker et al., 2020).

### Academic Integration

The studies analyzed in the university context either use the concept of adaptation or integration to refer to the process experienced by students when starting higher education, which depends on the existing personal characteristics when they start university, the quality of the institution hosting them, and the interaction established among their peers and institutions. This stage is assumed like the process of adjustment lived by students with their immediate surroundings, with their learning process, and the commitment toward the educational institution where they study (Matta et al., 2017). In this kind of process, it is important to consider the intensity and quality of relationship established between students and their classmates, teachers, administrative staff, and authorities, as well as the possibility to participate in extracurricular activities. The academic integration can be understood as “a student’s potential to benefit from academic experiences [which] requires that the student is able to meet the institution’s educational demands and that the institution is able to meet the student’s educational desires” (Clark et al., 2014, p. 31).

Current studies have shown that motivation is positively related to the academic integration perceived by students. These results indicate that experience in the sense of choice and personal will to be involved in learning activities foster adaptation to the university life that enables the academic performance and continuity of studies (Clark et al., 2014; van Rooij et al., 2018). On the other hand, studies show a significant relationship between basic psychological needs and the adaptation of students to university life. More specifically, it has been observed that higher levels of satisfaction of basic psychological needs are significantly related to high levels of academic integration, which favors both academic success and performance (Raižienë et al., 2017; Mohamedhoesein and Crul, 2018).

Furthermore, the empirical evidence shows that academic integration has presented a mediating effect on academic performance (Clark et al., 2014). In this regard, it has been observed that academic integration acts as a mechanism through which motivational factors can exert their influence on the achievement of academic results. Therefore, it is proposed that the academic integration experience can intervene the relation between the satisfaction of basic psychological needs and the intrinsic motivation, since the positive adjustment of the students in the degree program could promote the sense of choice and the interest to be involved in the learning activities.

According to the review of the evidence associated with the Chilean educational context, a lack of studies aiming at analyzing the psychological processes affecting the relationships between motivational factors facilitating the development of learning is observed.

### The Present Study

The aim of the study was to investigate the mediating role of academic integration on the relationship between the basic psychological needs satisfaction and the intrinsic motivation of Chilean university students. According to the review of the theoretical and empirical background, the following hypothesis was raised:

H_1_: Academic integration has a partial mediator effect on the relationship between the basic psychological needs satisfaction and intrinsic motivation. Specifically, the perception of the academic integration can intervene partially the link between the basic psychological needs satisfaction and the intrinsic motivation.

## Materials and Methods

### Research Design

The research design was quantitative, cross-sectional, and nonexperimental. An explanatory associative strategy was used, since relationship models according to an underlying theory were assessed (Ato et al., 2013).

### Participants

Based on a convenience sampling method, a total of 580 first-year students (61.9% female and 38.1% male) from a university in northern Chile were involved in the study. Participants were from 18 to 31 years old (*M* = 19.54, *SD* = 1.75). Among these participants, the highest percentage (31.9%) studied a degree program from the Faculty of Health Sciences, 20.3% were studying a degree program from the Faculty of Engineering, and 15.2% were studying a degree program from the Faculty of Education. Moreover, 53.3% of the students came from schools of the private subsidized type, 34.4% came from municipal schools, and 12.4% came from private schools. For the selection of the sample, the following inclusion criteria were considered: (a) students with active enrollment and (b) first-year students. On the other hand, the following exclusion criteria were considered: (a) students from external universities and (b) students with <70% attendance. Finally, to calculate the sample size required to the SEM analysis, the following criteria were considered: (a) an expected effect size of 0.5, (b) a desired statistical power level of 0.9, (c) 3 latent variables, (d) 19 observed variables, and (e) an alpha level of 0.05. The results showed that the minimum required sample size is 256 students, which indicates that the study sample size is adequate.

### Measurements

#### Basic Psychological Needs Satisfaction

The basic psychological needs satisfaction was measured using the Basic Psychological Needs Satisfaction and Frustration Scales (BPNSFS) (Chen et al., 2015). This instrument consists of 24 items grouped into 6 factors that measure satisfaction and frustration of each of the basic psychological needs proposed by the self-determination theory: (a) autonomy, (b) competence, and (c) relatedness. An example of an item is, “I feel like I can do things well.” A version adapted to the Spanish language in a sample of Chilean university students (Del Valle et al., 2018) was used. The items were evaluated on a 7-point Likert scale that ranged from 1 (totally disagree) to 7 (totally agree). In this study, the mean score of 12 items corresponding to the measure of satisfaction of each basic psychological need was calculated. The highest value indicates the highest degree of satisfaction. In the current study, the composite reliability coefficient for each factor was as follows: (a) autonomy = 0.61, (b) competence = 0.77, and (c) relatedness = 0.75. Moreover, the confirmatory factor analysis showed an adequate fit of the measurement model: χ^2^ (51) = 71.99, *p* = 0.03; Tucker Lewis Index (TLI) = 0.97; Comparative Fit Index (CFI) = 0.98; Root Mean Square Error of Approximation (RMSEA) = 0.03; and Root Mean Square Residual (SRMR) = 0.03.

#### Intrinsic Motivation

Intrinsic motivation was measured using the Academic Self-Regulation Scale (Vansteenkiste et al., 2009). It consists of 16 items that measure the reasons for getting involved in academic activities. The items are divided into four factors: (a) intrinsic regulation (four items, e.g., “Because it’s fun”), (b) identified regulation (four items, e.g., “Because I want to learn new things”), (c) introjected regulation (four items, e.g., “Because would I feel guilty if I did not study”), and (d) external regulation (four items, e.g., “Because I am supposed to do it”). A version applied in the Spanish language (Vergara-Morales et al., 2019) was used. In this study, it was considered the intrinsic regulation factor with its latent variables. The items were answered on a 7-point Likert scale that ranged from 1 (totally disagree) to 7 (totally agree). In the current study, the McDonald’s Omega coefficient was 0.81. Furthermore, the confirmatory factor analysis showed an acceptable fit of the measurement model: χ^2^ (2) = 1.28, *p* > 0.05; TLI = 1.01; CFI = 1.00; RMSEA = 0.00; SRMR = 0.01.

#### Academic Integration

Academic integration was measured using the Academic Experiences Questionnaire (Almeida et al., 2002). It consists of 60 items that evaluate the experiences and feelings of the students regarding the quality of academic experiences in coping with the university context. The items are divided into five factors: (1) personal, which evaluates students’ perceptions regarding their physical and psychological well-being; (2) interpersonal, which measures the relationship between peers; (3) degree program, which evaluates adaptation to undergraduate; (4) study, which assesses the competencies for the study of learning content; and (5) institutional, which measures the interest of students toward the academic institution. A Spanish language version adapted in the Chilean university context was used (Abello et al., 2012). In this study, the factor (3) was considered since it constitutes experiences that imply the students’ relationship with the academic context at the degree program level. The items were answered on a 7-point Likert scale that ranged from 1 (totally disagree) to 7 (totally agree). In the current study, the McDonald’s Omega coefficient was 0.84. Furthermore, the confirmatory factor analysis showed an acceptable fit of the measurement model: χ^2^ (152) = 142.64, *p* < 0.01; TLI = 0.91; CFI = 0.93; RMSEA = 0.06; and SRMR = 0.04.

### Procedure

The research was authorized by the Ethics Committee of a university in northern Chile. During the academic time, students were asked to complete the self-report questionnaires within the classroom, considering the supervision of a trained research assistant. Before responding, the students signed an informed consent letter. The data collection process was guided by the ethical considerations of the American Psychological Association (APA) and the ethical principles defined for research with human beings (Acevedo, 2002).

### Data Analyses

First, the descriptive data analysis was performed by calculating the mean, standard deviation, and the values of skewness and kurtosis, considering a cutoff point of ±2 to indicate acceptable values (Ryu, 2011). Moreover, Pearson’s correlation coefficient was used to assess the degree of association between the study variables. Second, the structural equation modeling analysis was performed using the maximum likelihood (ML) estimation method, and the goodness of fit was evaluated considering the following indexes and criteria: chi-square (χ^2^) and normed chi-square (χ^2^/df) = values <2 (Ullman, 2001) and <5 (West et al., 2012) are considered acceptable; TLI and CFI values ≥0.90 indicate an acceptable fit, and values ≥0.95 indicate a good fit; RMSEA and SRMR values ≤0.50 indicate a good fit, and values ≤0.80 indicate an acceptable fit (Schweizer, 2010). Finally, the bootstrapping procedure with 10,000 iterations was used to evaluate direct and indirect effects, which were considered statistically significant if the 95% confidence interval estimates did not contain the value of zero (Hayes, 2018). Data analyses were performed using SPSS 21 (IBM Corporation, 2012) and Mplus 8 (Muthén and Muthén, 2012).

## Results

### Descriptive and Correlational Analysis

Table 1 shows that BPNS presented the highest mean score (*M* = 6.03), followed by IM (*M* = 5.76). Moreover, it is observed that the data presented adequate levels of dispersion, since the standard deviation values were close to 1.00. In addition, the skewness and kurtosis values indicate a normal trend of the data, since they are in the range ±2. Regarding bivariate relationships, it is observed that the BPNS was positively associated with IM (*r* = 0.42, *p* < 0.01) and AI (*r* = 0.47, *p* < 0.01). Therefore, it is inferred that while students perceive high levels of basic psychological needs satisfaction, high levels of intrinsic motivation and academic integration are observed.

**TABLE 1:** Descriptive statistics and bivariate correlations between study variables.

	**M**	**SD**	**S**	**K**	**1**	**2**	**3**
1. BPNS	6.03	0.59	–0.41	–0.35	1.00	0.42**	0.47**
2. IM	5.76	1.06	–1.28	2.02		1.00	0.65**
3. AI	5.41	0.76	–0.91	0.88			1.00

BPNS, basic psychological needs satisfaction; IM, intrinsic motivation; AI, academic integration. **p < 0.01.

### Mediation Analysis

**FIGURE 1:**
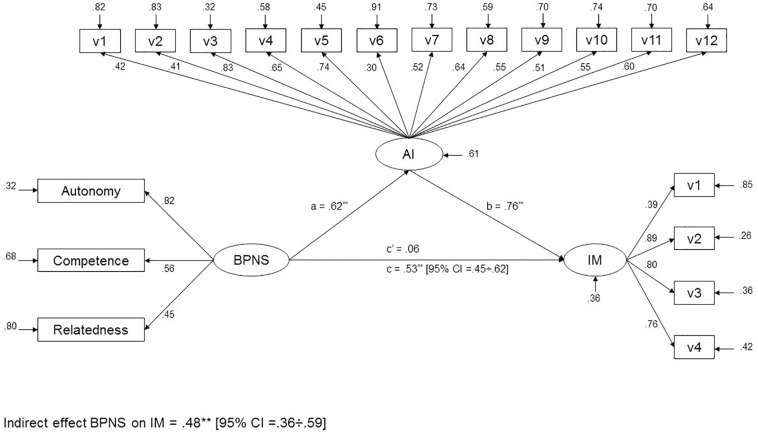
Single mediation model. BPNS, basic psychological needs satisfaction; AI, academic integration; IM, intrinsic motivation; **p < 0.01; standardized coefficients.

The proposed model contained 3 latent factors and 19 indicators (see Figure 1): 1 factor that reflects the basic psychological needs satisfaction at the university using 3 indicators; 1 factor that indicates the measure of academic integration with 12 indicators; and 1 factor that reflects intrinsic motivation using 4 indicators. Results showed that this model meets the recommended standards and evidenced an acceptable fit to the observed data, explaining 39% of the academic integration variance and 64% of the intrinsic motivation variance: χ^2^ = 491.88, χ^2^/df = 3.30, TLI = 0.90, CFI = 0.91, RMSEA = 0.06, and SRMR = 0.05.

Considering the good fit of the hypothesized model, direct and indirect effects were calculated (Figure 1). First, the BPNS is significantly related to AI (*a* = 0.62, *p* < 0.01). Second, AI is significantly related to IM, after controlling for the BPNS (*b* = 0.76, *p* < 0.01). Third, the BPNS is significantly and indirectly associated with IM through AI (*ab* = 0.48, *p* < 0.01, 95% CI = 0.36/0.59). Since the direct effect of BPNS on MI was not significant (*c*′ = 0.06, *p* > 0.05), the Wald test to check if the direct effect is equal to 0 was used. In this regard, the results showed that the Wald test was significant (*W* = 78.94, *p* < 0.01), which indicated the existence of the direct effect of BPNS on MI. Therefore, a partial mediator role for AI on the relationship between BPNS and IM is concluded.

## Discussion

The aim of the current study was to investigate the mediating role of academic integration on the relationship between the basic psychological needs satisfaction and the intrinsic motivation of Chilean university students. Consistent with the self-determination theory (Deci and Ryan, 2000), a positive and significant association among the basic psychological needs satisfaction, academic integration, and intrinsic motivation was observed. These results indicate that when students perceive teaching and learning environments that facilitate the necessary conditions to satisfy the basic psychological needs, not only they activate their interest, effort, and sense of choice to participate in the learning activities but also they experience a positive adaptation to the university life. These results are consistent with the findings of the studies by Raižienë et al. (2017), Pulyaeva and Nevryuev (2020), and Walker et al. (2020).

Moreover, it was identified that the basic psychological needs satisfaction had an indirect effect on the intrinsic motivation of the students through academic integration. Specifically, it was found that the academic integration had a partial mediating effect on the relationship between the basic psychological needs satisfaction and the intrinsic motivation, which supports the fulfillment of the research hypothesis. Unlike the findings of the study by Clark et al. (2014), it was observed that academic integration had a positive effect on intrinsic motivation. This difference can be supported by way of measuring academic integration since this study focused on adaptation and identification with the degree program. In this regard, it was observed that when students perceive a positive experience of integration at the degree program level, their interest and personal satisfaction with learning activities increase. Therefore, academic integration benefits the development of students’ intrinsic motivation for the development of learning.

The intrinsic motivation is a key psychological process for students to achieve quality learning, since it involves “activities done for their own sake,” or for their inherent interest [which] are not dependent on external incentives or pressure, but rather provide their own satisfactions and joys” (Ryan and Deci, 2020, p. 2). Intrinsically motivated students engage in learning activities from their own will and interest, since their actions are based on the interests, preferences, and desires that guide the decision to engage in academic activities (Reeve, 2010). In this regard, studies highlight the influence of intrinsic motivation on the quality and quantity of knowledge, skills, and competencies involved in the teaching-learning process (Koludrović and Ercegovac, 2015).

Based on the findings, the influence of the basic psychological needs satisfaction on the development of intrinsic motivation of university students is observed. In this regard, they facilitate the development of the sense of choice and personal will of students to be involved in academic activities, which supports the achievement of learning. However, it is also observed that academic integration, understood as the degree of adjustment to the educational environment, not only activates interest, pleasure, and satisfaction to get involved in learning activities but also can help the development of intrinsic motivation. Hence, students experiencing high degrees of adaptation in the university context “can adjust their own psychological resources to make themselves suitable for the new environment and changing conditions” (Zhou and Lin, 2016, p. 5). The results indicate that the mediating role of the academic integration facilitates the development of positive levels of intrinsic motivation, which promotes the activation of the sense of choice and the personal will of students to get involved in the academic activities. Therefore, it was observed that the perception of balance between the educational demands of the degree program and the satisfaction of educational desires explains the relationship between the basic psychological needs satisfaction and intrinsic motivation, since the academic integration implies a feeling of belonging to the degree program that constitutes a need related to the adaptation of students into the academic process.

The research findings suggest that students not only need to perceive the basic psychological needs satisfaction to facilitate the development of intrinsic motivation, but they also need a positive experience of academic integration. Therefore, the basic psychological needs satisfaction and academic integration are important factors for the intrinsic motivation of university students. Furthermore, the findings suggest that to facilitate the development of intrinsic motivation in the teaching-learning process, it is not only important that the teaching environments facilitate the educational conditions necessary to satisfy the basic psychological needs, but it is also important that higher education institutions promote actions to support the academic integration of university students.

Currently, most higher education institutions are working to implement programs that allow students to develop and maintain motivation for university studies since their direct influence on academic performance has been recognized. In this regard, to include intervention plans and specific lines of action for students to have a positive process of adaptation to life and university experiences is essential to maintain intrinsic motivation. Moreover, it is significant to consider that the satisfaction of the psychological needs of autonomy, competence, and relationship should not only be promoted in students but also should be considered as fundamental variables to be included in the curricular designs and pedagogical activities carried out by teachers.

### Limitations

First, the limitations of the study are related to the characteristics of the sample, since it was concentrated on university students. It is important that future research studies consider a more heterogeneous sample that allows the validity of the findings to be expanded. Second, the study was carried out considering a cross-sectional design, making the interpretation of the causal inference difficult. Thus, it is important that further studies consider longitudinal research designs.

## Data Availability Statement

The raw data supporting the conclusions of this article will be made available by the authors, without undue reservation.

## Ethics Statement

The studies involving human participants were reviewed and approved by the Comité de Ética de Investigación Científica, Universidad de Antofagasta, Chile. The patients/participants provided their written informed consent to participate in this study.

## Author Contributions

JV-M conceived the idea, performed the statistical analyses, and wrote the main part of the manuscript. MD built the conceptual structure and wrote part of the manuscript. Both authors critically read the work and approved the final draft of the manuscript.

## Conflict of Interest

The authors declare that the research was conducted in the absence of any commercial or financial relationships that could be construed as a potential conflict of interest.
